# Non-invasive control interfaces for intention detection in active movement-assistive devices

**DOI:** 10.1186/1743-0003-11-168

**Published:** 2014-12-17

**Authors:** Joan Lobo-Prat, Peter N Kooren, Arno HA Stienen, Just L Herder, Bart FJM Koopman, Peter H Veltink

**Affiliations:** Department of Biomechanical Engineering, University of Twente, Drienerlolaan 5,Enschede, 7522 NB The Netherlands; Department of Physics and Medical Technology, VU University Medical Center, Van der Boechorststraat 7,1081 BT Amsterdam, The Netherlands; Department of Physical Therapy and Human Movement Sciences, Northwestern University, 645 N. Michigan Ave. Suite 1100, 60611 Chicago, IL USA; Department of Precision and Microsystems Engineering, Delft University of Technology, Mekelweg 2, 2628 CD Delft, The Netherlands; Department Mechanical Automation and Mechatronics, University of Twente, Drienerlolaan 5, 7500 AE Enschede, The Netherlands; Department of Biomedical Signals and Systems, University of Twente, Drienerlolaan 5, 7500 AE Enschede, The Netherlands

**Keywords:** Non-invasive control interface, Active movement-assistive devices, Motion intention detection, Biomechatronics, Human movement control system

## Abstract

Active movement-assistive devices aim to increase the quality of life for patients with neuromusculoskeletal disorders. This technology requires interaction between the user and the device through a control interface that detects the user’s movement intention. Researchers have explored a wide variety of invasive and non-invasive control interfaces. To summarize the wide spectrum of strategies, this paper presents a comprehensive review focused on non-invasive control interfaces used to operate active movement-assistive devices. A novel systematic classification method is proposed to categorize the control interfaces based on: (I) the source of the physiological signal, (II) the physiological phenomena responsible for generating the signal, and (III) the sensors used to measure the physiological signal. The proposed classification method can successfully categorize all the existing control interfaces providing a comprehensive overview of the state of the art. Each sensing modality is briefly described in the body of the paper following the same structure used in the classification method. Furthermore, we discuss several design considerations, challenges, and future directions of non-invasive control interfaces for active movement-assistive devices.

**Electronic supplementary material:**

The online version of this article (doi:10.1186/1743-0003-11-168) contains supplementary material, which is available to authorized users.

## Introduction

**Figure 1:**
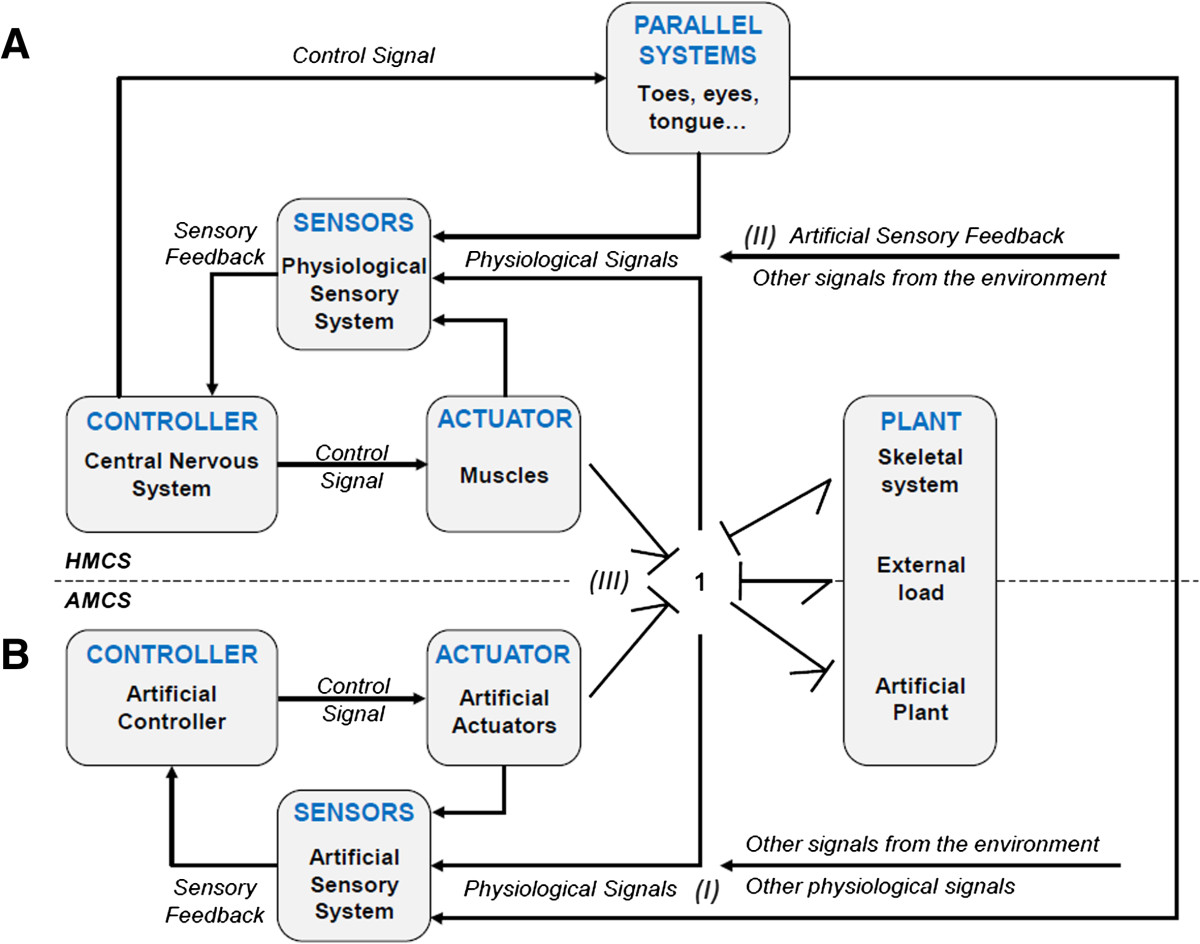
**Schematic block diagram of the Human Movement Control System (A) in parallel with the Artificial Movement Control System (B).** Three kinds of interactions between the HMCS and AMCS can be distinguished: (I) detection of the motion intention of the user; (II) provision of feedback to the user regarding the state of the AMCS, the HMCS or the environment; and (III) exchange of mechanical power between plants. Both the human and the artificial systems are depicted as dynamic systems in which both the human muscles and artificial actuators generate forces to transfer power and hence move the combined plant composed of the mechanical structure of the assistive device, the human musculoskeletal system and the environment (depicted as “external load”). Note that the interaction between the actuators and the plant is pictured with a *bond graph* that represents the energy exchange between them (i.e., movement and force in this particular case). The power 1-junction states a common velocity of all components. The reader is referred to [3] for further information on bond graphs. Modified from [1].

The ability to move in a controlled and stable manner is an essential trait for the human body. The Human Movement Control System (HMCS) can be modeled as in Figure 1A. The HMCS consists of a mechanical structure, *the plant*, which represents the skeleton and passive tissues, the *actuators*, which represent the muscles, and a *controller*, which represents the central nervous system and receives sensory feedback from the physiological *sensors*[1, 2]. Movement impairments, due to disease or trauma, can occur at various levels of the HMCS, affecting one or several components of the system: blindness affects the “sensors,” while muscular dystrophy affects the “actuators.”

Advances in neuroscience, engineering, and computer science have led to an acceleration in the development of biomechatronic systems, capable of actively assisting the impaired motor functions of patients affected by neuromusculoskeletal disorders [2, 4, 5]. Assistive devices have been classified by the International Organization for Standardization (ISO) in the standard ISO 9999:2011 according to their main function. Artificial Movement Control Systems (AMCSs) function in parallel to the impaired HMCS and can be modeled with the same components as the HMCS: a *plant* representing the mechanical structure and passive elements, such as springs or dampers, and an artificial *controller* that receives the data measured from the *sensors* and generates control signals to operate the *actuators* (Figure 1B).

Three kinds of interactions between the HMCS and the AMCS can be distinguished [2]: (I) detection of the movement intention of the user; (II) provision of feedback to the user regarding the state of the AMCS, the HMCS or the environment; and (III) exchange of mechanical power between both plants. Note that providing feedback to the user is especially relevant when the human sensory system is disturbed.

Several physiological phenomena occur in every subsystem of the HMCS. Some of these phenomena can be measured and associated to the motion intention of the user and therefore can be exploited for the effective control of an AMCS. Neural signals from the central nervous system, neural activation of the muscles, muscle contraction forces and small movements and forces of the human plant are some examples of signals that are *implicitly* related to the motion intention. Motion intention can also be derived from *explicit* commands of the user, for example by pressing command switches, through speech, or through head, tongue or eye movements. Explicit commands are generated by *Parallel Systems*, which are defined as systems that function in parallel to the supported system (see Figure 1A).

Researchers have explored a wide variety of invasive and non-invasive methods to derive the user’s movement intention. However, a comprehensive overview of these methods is not available, which hampers efficient and well-founded selection of a suitable control interface for a given application. To illustrate the wide spectrum of strategies, this paper presents a comprehensive review of non-invasive sensing modalities for motion intention detection in active movement-assistive devices. The strategies are classified in a systematic way that provides a clear overview of the state of the art and a framework in which new sensing modalities can be included. This review is limited to non-invasive interfaces (specifically meaning that the sensors are not implanted in the human body) as these are easier to use with a wide variety of patients [6–8].

The paper is structured as follows. Review section introduces the classification method used to categorize the control interfaces, briefly describes each of the interfaces and discusses several design considerations. Finally, Conclusions section presents our conclusions and future directions.

## Review

### Classification method

**Figure 2:**
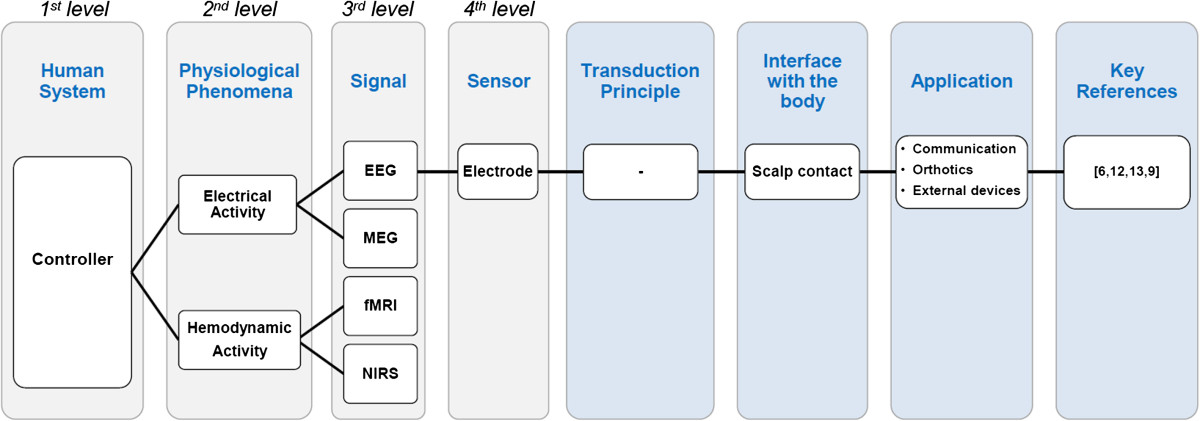
**Classification Method.** Example of the classification method illustrated with a schematic block diagram. See Table 1 for a full overview.

The inventory of control interfaces for motion intention detection resulting from the literature search was stratified through a four-level classification (see Table 1 and Figure 2). The first level was defined according to the subsystems of the HMCS (controller, actuators, plant and parallel systems), and the second level was defined according to the physiological phenomena that occur in every subsystem. The set of signals that can be measured for every physiological phenomenon defines the third level of classification, and the sensors used to measure these signals define the fourth level. For each sensor/signal, its transduction principle, interface with the body, area of application, and key references were indicated. Note that the inventory of control interfaces using Parallel Systems is illustrative and not complete and has been added as an additional subsystem of the HMCS in the first level of the classification.

**Table 1:** **Stratified inventory of non-invasive control interfaces used for motion intention detection in active movement-assistive devices**

Human system	Physiological phenomena		Signal	Sensor	Transduction principle	Interface with body	Application	Key reference
		Electric current	EEG	Electrode	-	Skin contact	C/P/O/E	[9–14]
			MEG	MEG machine	Induction	No contact	C/O	[15, 16]
Controller	Brain activity	Hemodynamics	fMRI	MRI machine	Induction	No contact	C/E	[17, 18]
			NIRS	Spectrometer	Photoelectric	Near-infrared illumination of the brain	C/E	[19, 20]
	Muscle activation	Electric current	EMG	Electrode	-	Skin contact	O/P/E	[21–24]
						Targeted muscle reinnervation*	P	[25–27]
		Vibration	MMG	Microphone	Induction Piezoelectric	Skin contact	P	[28–31]
				Accelerometer	Piezoelectric	Skin contact	P	[30–32]
				Hall-effect sensor	Induction	Magnet on the skin	P	[33, 34]
			MK	Pneumatic sensor	Resistive Capacitive	Skin contact	P	[35, 36]
Actuators	Muscle contraction	Dimensional change		Encoder	Photoelectric	Skin contact	O	[37]
			SMG	Ultrasound scanner	Piezoelectric	Skin contact	P	[38–40]
			Electric impedance	Electrode	-	Electric current to skin	P	[41]
		Radial force and stiffness	MT/MK	Force-sensitive resistor	Resistive	Skin contact	O/P	[42–44]
				Piezoelectric transducer	Piezoelectric	Skin contact	O/P	[45]
		Force	Deformation	Strain gauges	Resistive	Tunnel muscle cineplasty*	P	[46]
		Hemodynamics	NIRS	Spectrometer	Photoelectric	Near-infrared illumination of the muscle	P	[47–50]
		Body segment movement	Body segment movement	IMU	Piezoelectric	Skin contact	P/O	[51, 52]
	Movement			Camera	Photoelectric	No contact	E	[53]
	Relative joint movement	Joint rotations	Goniometer	Potentiometer	Resistive	Skin contact	P/O	[54–57]
Plant				Bending sensor	Resistive	Skin contact	P/O	[58]
				Encoder	Photoelectric	Skin contact/No contact	P/O	[59]
	Force/Pressure		Deformation	Force/Torque sensor (strain gauges)	Resistive	No contact	P/O/E	[60–62]
				Pressure sensor (force-sensitive resistor)	Resistive	Skin contact	P/O/E	[56, 63–66]
	Eye movement		Corneal reflection	Video camera	Photoelectric	Near-infrared illumination of the cornea	P/E	[67]
			EOG	Electrode	-	Skin contact	P/E	[68, 69]
				Accelerometer	Piezoelectric	Skin contact	E	[70–72]
	Head movement		Inclination	Video camera	Photoelectric	No contact	E	[73]
Parallel systems				Ultrasonic sensor	Piezoelectric	Skin contact	E	[74]
	Tongue movement		Contact with palate/Movement	Induction coil	Induction	Ferromagnetic material at the tip of the tongue	E/C	[75–77]
	Speech		Sound	Microphone	Induction Piezoelectric	No contact	P/E	[78–80]
	Hand movement		Angle	Joystick (potentiometers)	Resistive	Skin contact	O/E	[81–83]

C: communication; P: prosthesis; O: orthosis; E: external devices; (*) indicates one-time invasive method.

### Interfacing with the controller: brain computer interfaces

Current noninvasive brain-computer interfaces (BCIs) derive the user’s movement intention from electrical and hemodynamic signals from the brain.

#### Electrical brain activity

Electroencephalography (EEG) and magnetoencephalography (MEG) are well-established non-invasive methods that measure the average dendritic currents of a large proportion of cells from the scalp [84]. Several brain signals have been used for BCIs, including slow cortical potentials, low-frequency changes in filed potentials (such as P300) and α and β rhythms. Furthermore, BCIs can exploit signals related to external sensory stimulation such as auditory or visual stimuli (i.e., evoked potentials), or voluntary mental activity, such as imagining a movement. Even though MEG provides a higher signal quality than EEG and does not require the attachment of scalp electrodes, the latter is portable (i.e. does not require a shielded room) and less expensive. Consequently, EEG-based BCIs are currently commercially available (e.g., by intendiX®, g.tec medical engineering GmbH, Schiedlberg, Austria) for personal use to operate spelling and domestic devices.

**Figure 3:**
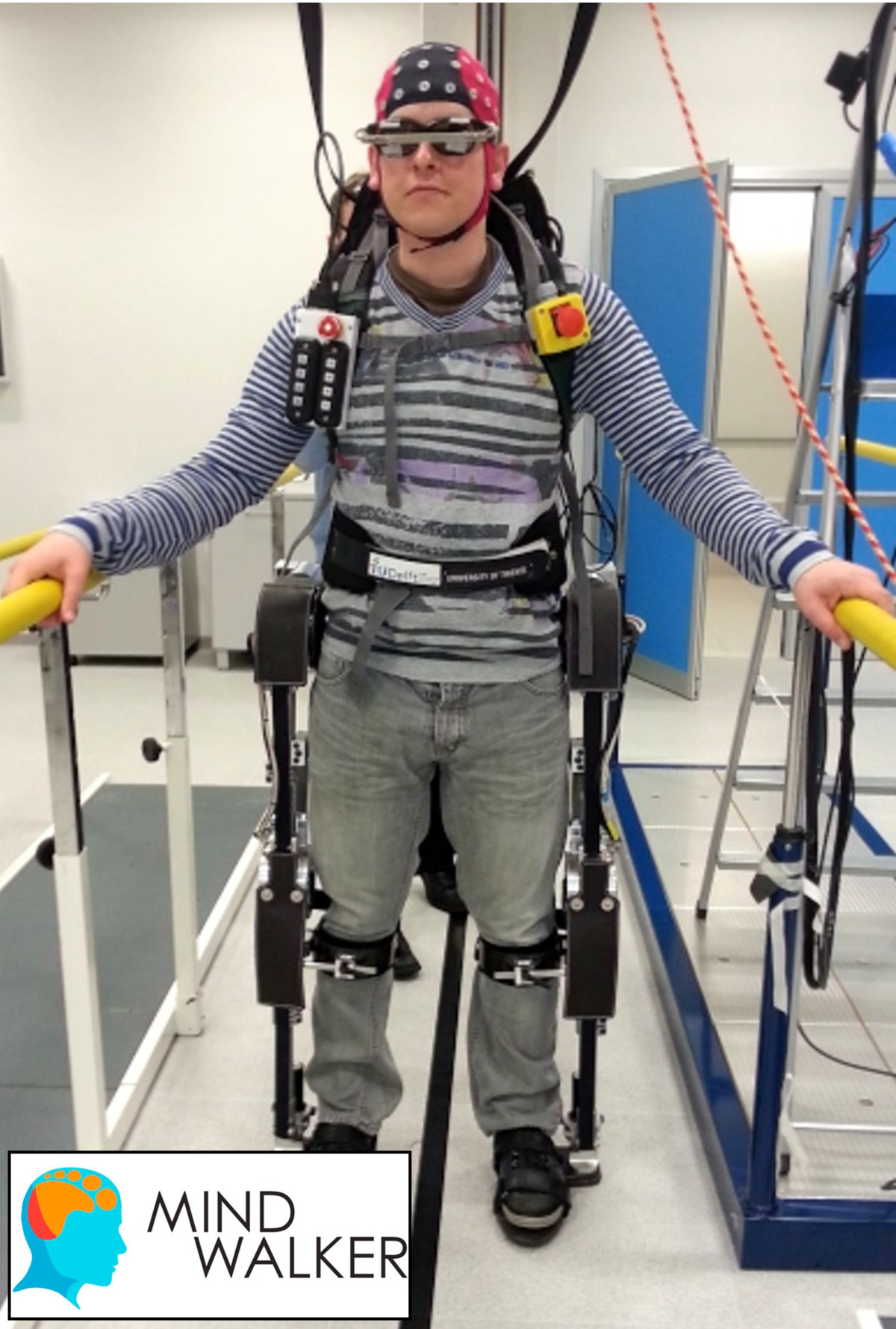
**EEG-based interface.** An EEG-based BCI used for the control of the Mindwalker lower-extremity exoskeleton [12, 87]. In this setup the BCI is controlled using Steady State Visually Evoked Potentials (SSVEP). The glasses that the user is wearing stimulate the retina with several flashing lights at different frequencies, and depending on which flashing light the users looks at, the brain will generate electrical activity at the same (or a multiple) frequency as the visual stimulus. With this method, different control states are assigned to the electrical brain signals with specific frequencies. Additional file 1 shows this EEG-based BCI controlling the Mindwalker lower-extremity exoskeleton. Figure courtesy of Mindwalker project.

While most current research on EEG- and MEG-based BCIs focus on providing basic communication control to people suffering from severe motor impairments [9, 15], researchers have also been exploring their capabilities for providing control of orthotic [12, 13, 16, 85–87] (see Figure 3 and Additional file 1), prosthetic [10], and external movement-assistive devices [8, 11, 88]. The main drawbacks of current EEG-based BCIs include the long training periods to learn to modulate specific brain potentials, the need to attach multiple electrodes to the scalp –both a time and cosmetic issue– the low information-transmission rate due to the filtering properties of the skull, low spatial resolution and high variability of the brain signals due to changes in background activity (e.g., motor, sensory, and cognitive activity) and learning processes [89, 90]. All these factors limit the applicability of BCIs as control interfaces of active movement-assistive devices. Today, BCI research is focused on the development of practical systems and their evaluation outside of the laboratory environment by end-users [14, 91, 92].

Additional file 1: **EEG-based interface.** An EEG-based BCI used for the control of the Mindwalker lower-extremity exoskeleton [12, 87]. In this setup the BCI is controlled using Steady State Visually Evoked Potentials (note the flickering lights in the inside part of the glasses). Video courtesy of Mindwalker project. (MP4 8 MB)

#### Brain hemodynamics

Beyond electric activity, hemodynamic signals from the brain are also used in BCIs. These signals are measured with functional magnetic resonance imaging (fMRI) or near infrared spectroscopy (NIRS). Both methods rely on the measurement of the task-induced blood oxygen level-dependent (BOLD) response, which was proven to be strongly correlated to the electrical brain activity [93]. Most studies using fMRI- and NIRS-based BCIs focused on their application to neurofeedback [94–96], and only a few studies have aimed to develop interfaces for communication [17, 97, 98], cursor control [99], environmental control [19, 100], and external robotic arm control [18, 20]. As for EEG, NIRS is also portable and both less expensive and less cumbersome than fMRI. Furthermore, in contrast to fMRI, when using NIRS, subjects can be examined in a sitting or standing position without movement constraints. However, the depth of brain tissue that can be measured using NIRS is 10 to 30 mm, which limits the measurements to cortical regions [93]. While these functional imaging methods are promising for non-invasive recording of activity across the entire brain (or cortical regions in the case of NIRS) at high spatial resolution [93], they suffer from a very poor information transfer rate [101, 102] that limits their functionality. NIRS-based BCIs are still in the early phases of research and development, and therefore their future potential value as control interfaces remains uncertain [103].

### Interfacing with the actuators: muscle activation interfaces

The recording of the electrical signals from muscle activation is known as electromyography (EMG). From a biomechatronic perspective, the muscle can be conceived as a biological signal amplifier of the low-amplitude electric potential from the motor neurons. The large majority of active prostheses that exist today, including commercially available devices, are controlled using surface EMG signals [89, 104].

Commercially available myoelectric upper-extremity prostheses are generally operated using proportional or on/off controls by measuring EMG from two independent residual muscles, or by distinguishing different activation levels of one residual muscle. Switching techniques such as muscle co-contraction or the use of mechanical switches or force-sensitive resistors are commonly implemented for enabling the sequential operation of several degrees of freedom (DOF) [89, 104, 105]. Lower-extremity prostheses and upper- and lower-extremity orthoses are commonly controlled estimating the joint angles or torques from EMG signals of muscles that mainly contribute to the supported motion [21, 22, 106, 107].

EMG-based control interfaces are widely used because of its easy access and generation, and its direct correlation to the movement intention. An EMG-based interface presents several drawbacks: It requires significant signal processing before it can be used as control signal due to its broad bandwidth and low amplitude; many patients have difficulties generating isolated and repeatable contractions [89]; and finally, the filtering properties of the limb tissue and the movement of the skin beneath the electrode notably affect long-term recordings [108]. All these factors make the control challenging for the user. To overcome these limitations, researchers are developing muscle synergy-based algorithms, motor neuron spike train decomposition algorithms, and new surgical procedures.

The increasing number of DOF in active prosthetic and orthotic devices to achieve higher functionality has been pushing the scientific community to develop EMG-based control interfaces capable of controlling multiple DOF in an intuitive and natural way. The identification of interactions between multiple muscles, commonly known as muscle synergies, using pattern recognition or regression algorithms is showing promising results toward achieving natural multifunctional control of prosthetic and orthotic devices [109].

EMG pattern-recognition algorithms are based on the assumption that humans can generate different yet repeatable muscle-activation patterns that are associated to specific movements, such as different grasping configurations. During the training of the algorithm, each programmed movement is linked to a stable muscle-activation pattern that is described by a set of features. These features should be repeatable across trials of the same movement and discriminative between different movements [110]. Once the training is completed, the algorithm extracts features from windowed raw EMG data and classifies them into the programmed movements. Many different variations of each of these steps have been investigated [111] trying to find a suitable tradeoff between speed and performance [112]. Pattern-recognition algorithms recently became commercially available (Coapt LLC., Chicago, USA). This technique has the potential to eliminate the need for isolated muscle activation and allow for the control of multiple degrees of freedom [89]. However, an important limitation of pattern-recognition algorithms is that they are only capable of classifying movements in sequence and not simultaneously and lack proportional controllability, which limits user acceptance [110].

EMG regression algorithms, which are based on non- negative matrix factorization, artificial neural networks, or linear regressions, present a functional improvement since they allow for simultaneous and proportional control of multifunctional prostheses [105]. Regression algorithms require a training data set for which a continuous relationship between EMG signals and plant kinematics or dynamics is known. Several studies have reported proportional and simultaneous control of two or more DOF in upper-extremity prosthetic [23, 113, 114] and orthotic [24, 115] devices. An additional movie file shows an amputee operating an upper-extremity prosthesis using both simultaneous and sequential EMG-based control during functional tasks (see Additional file 2).Additional file 2: **Simultaneous and sequential EMG-based control.** An amputee controlling the elbow and hand movements of an upper-extremity prosthesis with simultaneous and sequential EMG-based control. Video reused from [178]. (MP4 10 MB)

**Figure 4:**
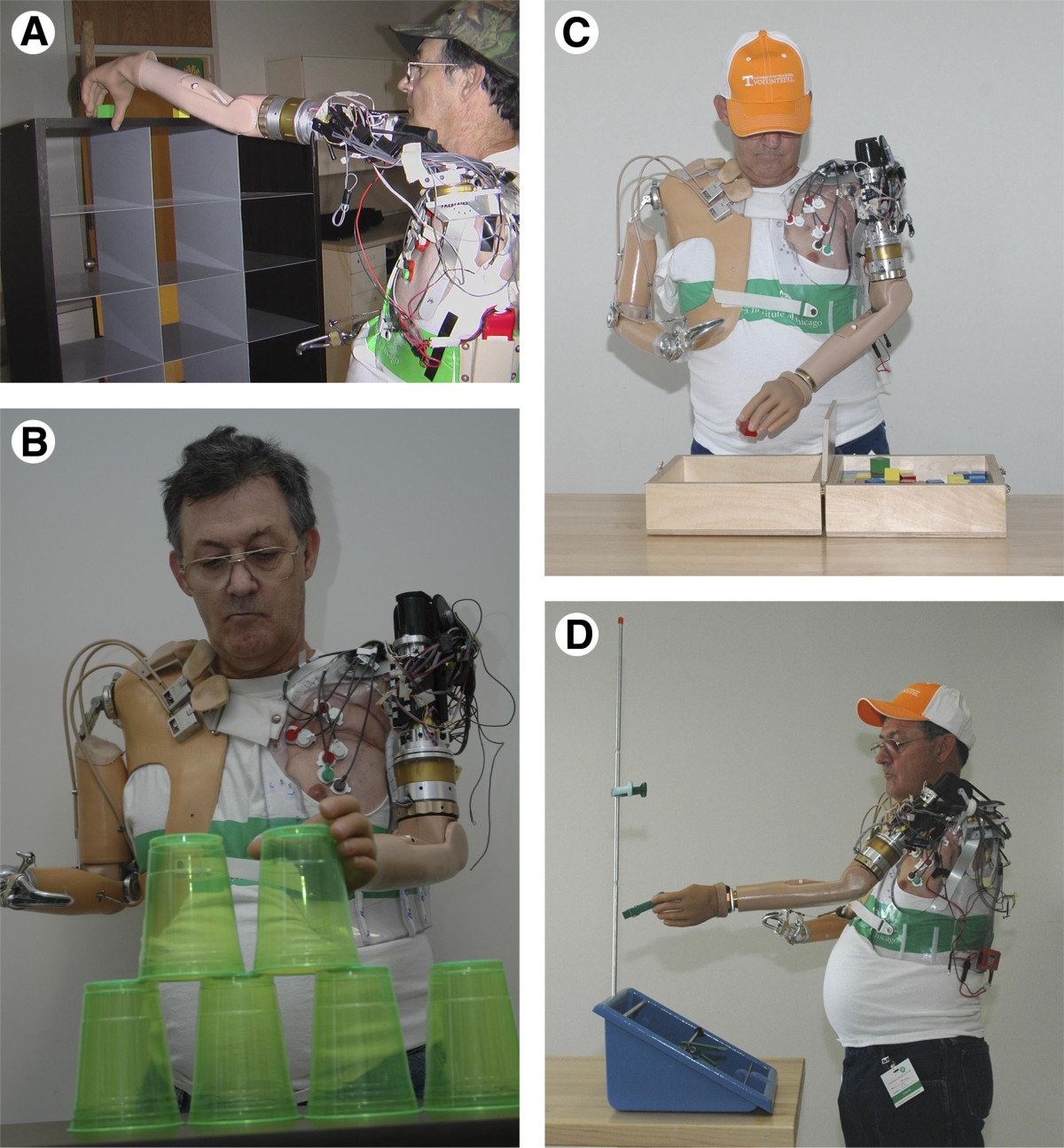
**EMG-based interface.** An amputated patient using a Targeted Muscle Reinnervation (TMR) EMG-based interface for the control of an active prosthetic arm [116]. With the TMR EMG-based interface, the patient could control a 6 DOF prosthesis consisting of shoulder flexion, humeral rotation, elbow flexion, wrist rotation, wrist flexion, and hand opening/closing control. The movement performance of this 6 DOF prosthesis (right arm) controlled with TMR EMG-based interface was evaluated and compared to the commercially available prosthesis (left arm) with 3 DOF (body-powered elbow and wrist rotation, and active terminal device) during several timed tasks: **A)** cubbies, **B)** cups, **C)** Box and blocks, and **D)** clothespin relocation task. The participant could control up to 4 DOF simultaneously, reach a larger workspace and perform some of the timed tasks faster using the TMR EMG-based interface. Figure reused with permission from Elsevier.

Targeted muscle reinnervation (TMR) is a surgical procedure developed by Kuiken et al. [25] that consists of rerouting the nerves that originally innervated the amputated limb to residual chest or upper-arm muscles (Figure 4). TMR is a one-time invasive method that allows a more intuitive control of a larger number of DOF [116] than standard EMG methods, since the prosthesis is controlled by EMG signals from the residual muscles that are activated by the nerves that previously controlled the amputated limb. Moreover, there is evidence that cutaneous sensory feedback of the amputated hand can be regained by reinnervating skin near or overlying the target muscles with residual afferent nerve fibers of the amputated hand [117]. TMR appears to be more suitable for high-level amputations [118] and current implementations still experience some difficulties separating the EMG signals from the different chest muscles. Recent studies aim at combining TMR with muscle synergies-based algorithms [26, 119], developing new targeted sensory reinnervation techniques [120], applying TMR for the control of lower-extremity prostheses [27, 121, 122], and implementing intramuscular EMG electrodes [123].

Finally, a recent approach that may lead to a new EMG-based control strategy is the use of high-density electrode grids that can extract spike trains of motor neurons and provide information on muscle-discharge patterns. The decomposition of EMG signals into spike trains of motor neurons can be used for proportional control of assistive devices and has shown a higher accuracy when estimating muscle force compared to conventional surface EMG recordings [110].

### Interfacing with the actuators: muscle-contraction interfaces

Several signals derived from the muscle-contraction phenomena have been used to detect motion intention: muscle vibration, dimensional change, radial muscle force and stiffness, longitudinal muscle force and muscle hemodynamics. The main advantage of muscle contraction-interfaces (MCIs) is that they are free from electromagnetic noise and may have a lower cost compared to EMG based interfaces. MCIs have only been used for the control of prosthetic devices.

#### Muscle vibration

The mechanical vibration that is generated when the muscles contract can be measured with microphones [28, 29], accelerometers [32], or a combination of both [30, 31]. This method is known as phonomyography (PMG), acoustic myography (AMG), or mechanomyography (MMG).

Orizio et al. reported a linear relationship between the root-mean-square (RMS) values of MMG signals recorded from the biceps brachii and the force of the contraction between 20 to 80 percent of the maximum voluntary contraction [124], which makes MMG potentially suitable for prostheses control. MMG offers several advantages over conventional EMG, including robustness to changes on skin impedance, less specific sensor placement, and reduced sensor cost [28, 30]. However, microphones and especially accelerometers are highly prone to limb-movement artifacts that compromises signal detection and classification [29]. To overcome this major problem, Silva and Chau [31] developed a coupled microphone-accelerometer sensor (embedded in silicone) that fuses data from both transducers to reduce dynamic noise. In their design, the accelerometer is used as a dynamic reference sensor to determine the source of the measured vibration (muscle contraction or limb movement). Recently, Posatskiy and Chau [125] developed a novel microphone with cylindrical and conical acoustic chambers that improves the robustness to limb-movement artifacts.

#### Muscle dimensional change

When the muscle contracts, dimensional changes also occur: the muscle shortens and consequently its cross-section area increases. The measurable signals resulting from this phenomenon are known as myokinemetric (MK) signals. MK signals have been measured with Hall-effect sensors, tendon-activated pneumatic (TAP) foam sensors, angular encoders, ultrasound scanners, and with electrodes that detect changes in electrical impedance.

Evidence from [33, 34] suggests that MK signals are inherently low in noise and that their magnitude can be directly used for control, avoiding any kind of signal processing. The study by Kenny et al. [33] developed a sensor that measured radial displacements of the muscle bulge with a Hall-effect transducer. They found that the MK signals of six upper-limb amputees could be generated with sufficient accuracy to perform a one-dimension tracking task with a low tracking error, and therefore had potential for the control of active upper-limb prostheses. However, it was also found that the MK signals were susceptible to socket slippage with time, as well as socket re-donning. The study by Heath [34] improved the sensor interface and proved the feasibility of using MK signals for proportional position control of prosthetic fingers.

The TAP sensors developed by Abboudi et al. [35] measured pressure differences at the skin surface generated by tendon displacements when finger flexor or extensor muscles contracted. TAP sensors presented a linear relation to finger force. Trials on three upper-limb amputees showed that the TAP sensors could provide effective control of individual finger movement and grasping. Since the performance of the TAP control interface is highly dependent upon accurate sensor placement and specific movement resolution at each sensor location, Curcie et al. [36] developed a pressure vector decoder able to discriminate specific finger flexion commands in real-time. This decoder decreased the dependence on sensor location, offering a more robust and reliable control of the TAP-based interface.

The study by Kim et al. [37] presents the development of a sensor that can measure muscle circumference changes using an angular encoder that was attached to an elastic arm band with a wire. The authors developed a calibration procedure and a biomechanical model to estimate the elbow torques from the measurements of the muscle circumference.

Recent studies propose the use of ultrasound scanners [38–40, 126] to measure changes in muscle thickness for the control of prosthetic devices. This method is known as sonomyography (SMG). SMG presents a linear relationship between ultrasound image features of the human forearm and the hand and wrist kinematic configurations [38, 127], suggesting that simple proportional control could be implemented for the operation of active movement-assistive devices. A recent study by González and Castellini [40] shows that a linear relationship also exists between specific image features and fingertip forces. Furthermore the study by Shi et al. [126] shows that SMG can be implemented in real-time using a two-dimensional logarithmic search algorithm. While it has been demonstrated that SMG could be potentially used for the control of active prosthetic devices, the current system used for the SMG measurements (i.e., standard ultrasound scanner) is not suitable for practical implementation because it is expensive and cumbersome [39].

Another signal derived from dimensional changes of the muscle was examined in the early 1970s by Kadefors and Olsson [41]. The study investigated electrical impedance as a measure of motion intent for the control of a robotic hand. The electrical impedance measured on the skin above a muscle varied when the dimensions of the muscle changed due to its contraction (among other factors). A recent study by Silva et al. [128] shows evidence that relates muscle activity and tissue resistivity changes and suggests that this information could be used for the control of assistive devices.

#### Radial muscle force and muscle stiffness

The stiffness of muscle tissue increases when it contracts. The measured force signals resulting from this phenomenon are known as myotonic (MT) [34] or myokinetic (MKT) [42] signals. These signals have been measured using arrays of force-sensitive resistors (FSRs) [42] or strain gauges [43, 129]. A recent study by Castellini and Koiva [44] used a tactile sensor placed between the arm and the table to measure changes in pressure distribution at the ventral side of the forearm while performing a variable force task with the fingertip. The authors report that the fingertip forces could be estimated with a high degree of accuracy from the pressure distribution measured at the forearm.

Recently, a study by Han et al. [45] presented a novel muscle stiffness sensor that could be worn over clothing, based on the measurement of the muscle resonance frequency. This sensor measured muscle stiffness by generating and sensing resonance vibrations using a piezoelectric transducer: As the muscle became stiffer, the resonance frequency became higher.

Muscle-stiffness-based interfaces are still in the early phases of research and development, and therefore their potential value as control interfaces for active movement-assistive devices remains unclear. None of the aforementioned studies reported results from experiments implementing this sensing modality in an assistive device and testing it with people with movement impairments.

#### Muscle force

**Figure 5:**
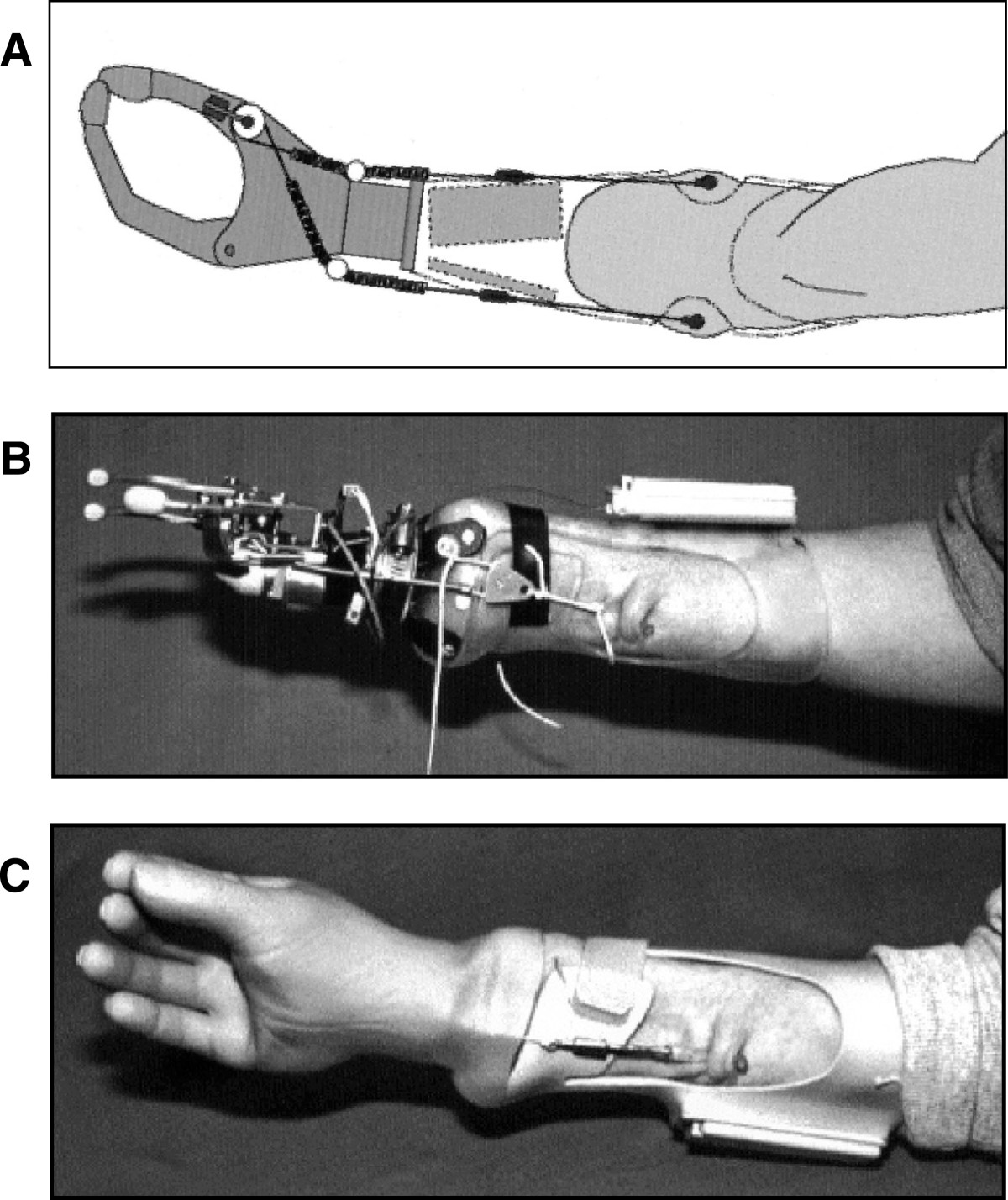
**Muscle-force-based interface.** The prosthesis is controlled by pulling on cables that mechanically link the tendons attached to the tunnel muscle cineplasty to a force transducer mounted to the thumb of the prosthetic hand. An artificial controller measures the tendon force produced by the muscle to operate the opening and closing of the hand. **A)** schematic representation of the prosthesis and the control interface, **B)** an early version of the prototype without a cosmetic hand glove, **C)** the final prototype of the prosthesis with a cosmetic hand glove. Figure modified from [46].

Direct muscle force control by muscle tunnel cineplasty (MTC; Figure 5) was first performed in the early 1900s, becoming popular after World War II [46, 130]. The major advantage of this one-time invasive method is that is capable of providing tactile and proprioceptive feedback from the terminal device back to the user complying with the concept of extended physiological proprioception (EPP) [46, 131]. However, such procedures lost favor in the 1970s due to the advent of clinically available myoelectric prostheses, which do not require any surgery. Moreover, MTC presented the disadvantage that patients sometimes lacked sufficient muscle force to power their prosthesis [130].

While traditionally MTCs provided both the control and the actuation of the prosthetic prehensor [132], Weir et al. [46] proposed a hybrid method where the MTC provided the control signal for the prosthetic prehensor, but the grasping force was supplied by an external power source. The authors of the study suggested that the implementation of multiple miniature forearm MTCs could be potentially used as control signals for independent multi-finger control. However, to the best of our knowledge, no further advancements on this method have been made up to date.

#### Muscle hemodynamics

NIRS has also been used to measure optical changes due to muscle contraction for the operation of active prostheses. Several physiological phenomena have been reported as responsible for the muscle optical changes, including blood perfusion [47, 133], muscle shortening [48], and muscle fiber direction [49]. Numerous studies show that NIRS signals are qualitatively similar to EMG signals in both magnitude and duration for different levels of contraction [47, 48, 50], with the particular difference that isometric and isotonic contractions can be distinguished from NIRS signals [49]. Furthermore, Herrmann et al. [133] proposed to combine EMG and NIRS signals to improve the pattern classification algorithm for the control of a prosthetic hand.

### Interfacing with the plant: movement interfaces

The human body moves as a result of the interaction between the forces generated by the muscles and the configuration of the skeletal system. Measurements of relative joint rotations and motion of body segments with respect to a fixed reference have been used to detect motion intention.

#### Body segment movement

Body segment motion has been measured with inertial measurement units (IMUs) and camera-based systems. In a study by Thomas and Simon [51] and in one by Moreno et al. [52] IMUs were used to control an active knee joint during walking. The study by Jiang et al. [53] presented a hand-gesture recognition interface system based on two Microsoft Kinect cameras (Microsoft; Redmond, Washington) for the control of external robotic arms. The interface was specifically developed for individuals with upper-level spinal cord injuries (SCIs) to perform a variety of simple object-retrieval tasks. One camera was used to interpret the hand gestures and locate the operator’s face for object positioning. The other camera was used to automatically recognize different daily living objects for test subjects to select. A similar interface was developed by Martin et al. [134] and used several infrared sensors to measure hand movements to control an active arm support for patients suffering from muscular weakness.

#### Relative joint movement

**Figure 6:**
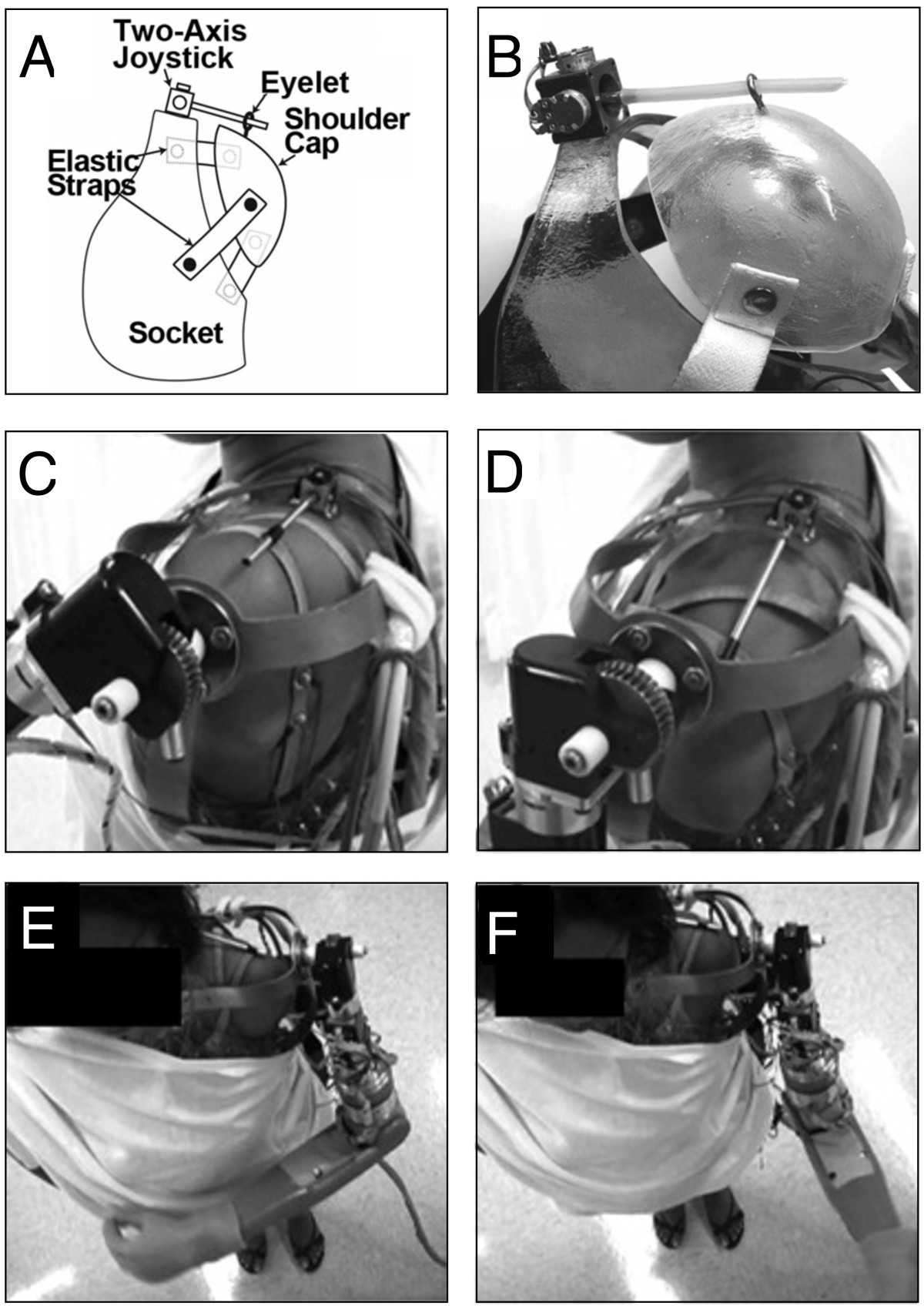
**Joint-rotation-based interface.** 2 DOF joystick used for the control of a prosthetic arm (shoulder flexion-extension, shoulder internal-external rotation) with the residual shoulder motion. **A)** Diagram of the control interface used to measure the residual shoulder motion, **B)** close-up of the prototype, **C)** shoulder elevation produces shoulder flexion, **D)** shoulder depression produces shoulder extension, **E)** shoulder protraction produces internal shoulder rotation, **F)** shoulder retraction produces external shoulder rotation. Additional file 3 shows an amputee using this shoulder-joystick-based interface to control the shoulder motions of an active prosthesis. Figure modified from [56].

Angular displacement between two adjacent body segments has been measured using electrogoniometers, that are attached to two adjacent body segments and produce an electrical signal proportional to the angle. Several kinds of electrogoniometers have been used to measure angular displacements. Early goniometers used simple angular potentiometers. Doubler and Childress [54] and Gibbons et al. [55] used these sensors to investigate the EPP concept in the control of active upper-extremity prostheses. Recent studies also investigated the performance of controlling prosthetic arms with the residual shoulder motion measured with a two-DOF joystick [56, 57] (see Figure 6 and Additional file 3). Although potentiometers can measure rotations about only one axis and the accuracy of the measurements depends on their alignment with the human joint, these sensors are still a common component in active movement-assistive devices [135, 136]. Another common solution for measuring angular displacement to control active prosthetic and orthotic devices is the use of angular encoders [59] or bending sensors [58, 137]. The control strategy developed by Wang et al. [138] estimates the location of the body center of mass in the sagittal and frontal plane using IMUs and angular encoders to measure the weight shift and trigger the stepping movement of a lower-extremity exoskeleton.

### Interfacing with the plant: force interfaces

**Figure 7:**
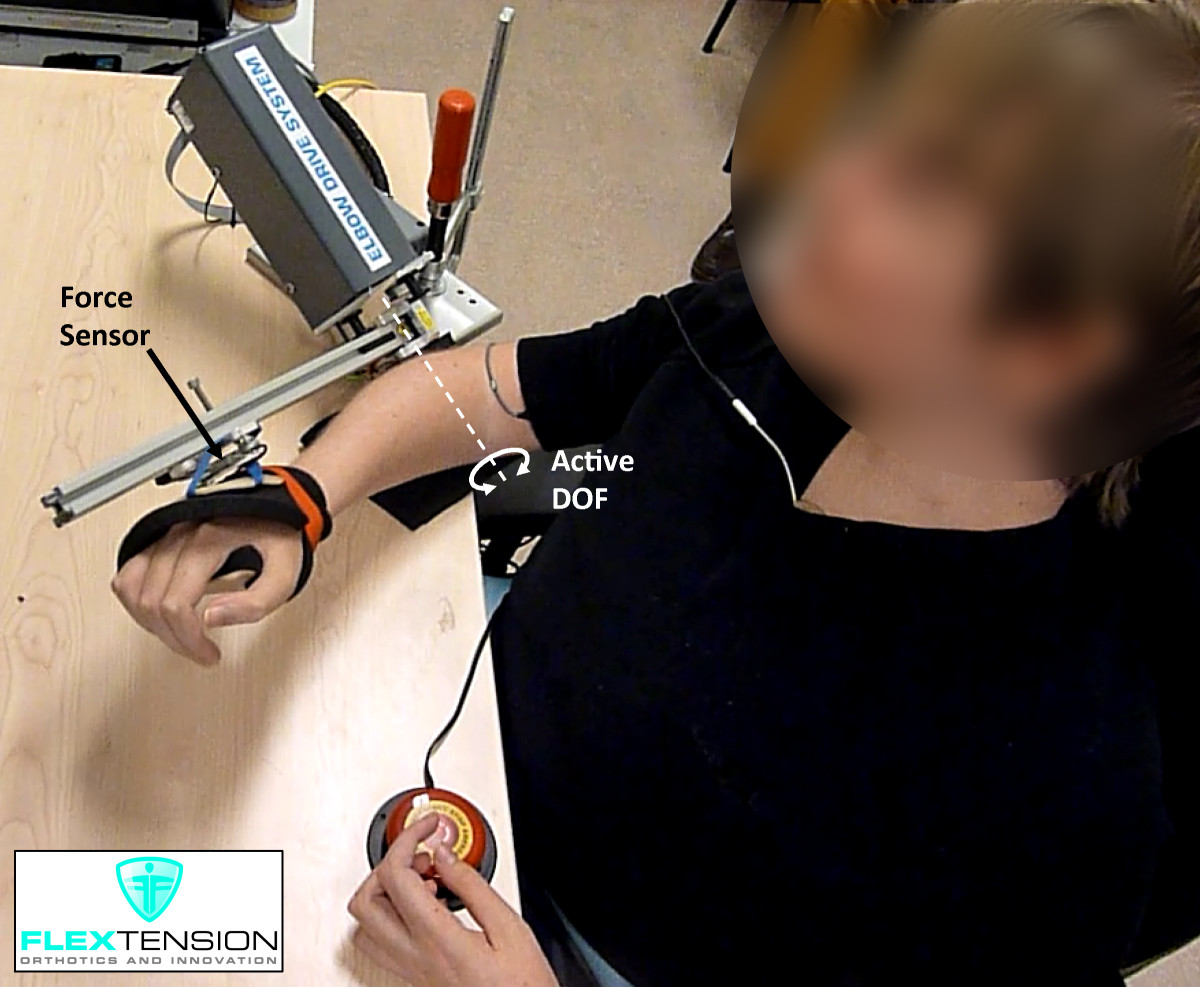
**Force-based interface.** An adult man with Duchenne muscular dystrophy with no arm function left using a force-based interface to operate an active elbow orthosis [62]. The force sensor measures the interaction force between the orthosis and the user, which is used as a control input for an admittance controller. A critical aspect for the usability of this interface is the accurate identification of the gravitational and joint stiffness forces (which are pose-dependent) required to distinguish the low-amplitude voluntary forces of the user. Additional file 4 shows the force calibration procedure used to identify the gravitational and joint stiffness forces. Additional file 5 shows a man with Duchenne muscular dystrophy performing a discrete tracking task using the force-based interface to control the active elbow orthosis. Figure courtesy of Flextension project.

The human plant can exert forces to the environment that can provide information about the motion intention of the user. Force-based interfaces have been implemented using force-torque sensors [60–62] or simple FSRs for the control of active upper-extremity orthoses [63–66] and prostheses [56]. These kind of interfaces generally implement control strategies where the output motion is related to the input force [139]. An advantage of force-based interfaces is that force sensors can be embedded in the mechanical structure of the assistive device, avoiding any preparation for the placement of the sensors on the user. Recently, Ragonesi et al. [140] reported measurements of gravitational and joint stiffness torques in patients with muscle weakness with the end goal of obtaining a robust model that could be used for the control of an active arm support. The authors found that voluntary forces of individuals with muscular weakness were very hard to measure since gravitational forces were much larger. Subject-specific models were suggested as a strategy to optimize the identification of voluntary forces. The study by Lobo-Prat et al. [62] demonstrated that an adult man with Duchenne muscular dystrophy with no arm function left, could successfully control an active elbow orthosis using the low-amplitude force (and EMG) signals that still remained measurable (Figure 7). In the aforementioned study, the gravitational and joint stiffness forces were measured during a calibration procedure in which the orthosis together with the relaxed forearm of the participant slowly moved across its range of motion. The gravitational and joint stiffness forces measured during this calibration procedure were subtracted from the actual measured force to compensate them. Additional movie files show an adult man with Duchenne muscular dystrophy with no arm function left performing the calibration procedure used to measure the gravitational and joint stiffness forces (see Additional file 4), and using the force-based control interface to perform a discrete position-tracking task (see Additional file 5).

Additional file 4: **Force calibration procedure.** A critical aspect for the usability of force-based interfaces in individuals with severe muscular weakness is the accurate identification of the gravitational and joint stiffness forces. This video shows the force calibration procedure used to measure the pose-dependent gravitational and joint stiffness forces of the participant. The absence of EMG activity shows that the participant was relaxed during the measurement. Video courtesy of Flextension project. (MP4 9 MB)Additional file 5: **Force-based interface.** An adult man with Duchenne muscular dystrophy with no arm function left using a force-based interface to operate an active elbow orthosis and performing a discrete position task. The plot in the bottom of the video shows the interaction force between the participant and the orthosis used as a control input for the admittance controller. The reader is referred to [62] for further information on the design and control of this active elbow support. Video courtesy of Flextension project. (MP4 17 MB)

In patients with severe movement and force limitations, it is very challenging to use movement- and force-based interfaces. These type of control interfaces are more often implemented in rehabilitation robots where patients need training to regain mobility and strength [107, 141].

### Interfacing with parallel systems

Apart from deriving the motion intention from signals that originate from the supported systems, several methods have been proposed that exploit signals from parallel systems such as the eyes, the mouth or the head to derive the movement intention of the user. This section reviews six groups of common interfaces that derive the intent of the user through signals generated by parallel systems.

#### Eye interfaces

Eye tracking systems are a common method for the control of spelling devices or computer cursors in patients with severe movement impairments. Several eye-trackers have been developed, including camera-based methods, which measure changes in corneal reflection while infrared light is projected to the eye [142], and electrical-based methods that measure the electrooculographic (EOG) potential from surface electrodes.

Duvinage et al. [68] proposed an innovative system based on EOG and a programmable central pattern generator to control a lower-limb prosthesis. The control method was composed of two steps: First, an EOG-based eye-tracking system generated high-level control commands (such as faster, slower, or stop), according to specific eye movement sequences executed by the user; and second, a pattern generator, following the high-level commands derived from the user’s eye motion, provided the low-level commands for the control of the actuators.

In the study by Chen and Newman [69], EOG was used to control two-dimensional movements of an external robotic arm that resembled the human arm configuration. Eye movement patterns such us saccades, fixation, or blinks were detected from the raw eye gaze movement data by a pattern-recognition algorithm and converted into control signals according to predefined protocols. The authors suggested that one option to extend the movement control to three-dimensional space was to switch between predefined action planes in which the EOG control would still be two-dimensional.

While eye movement interfaces proved to be very accurate in two-dimensional space, three-dimensional gaze-tracking is more challenging [143]. The three-dimensional gaze-tracking problem consists of mapping pupil coordinates for left and right eye to a three-dimensional point referenced to the user’s head coordinated. A recent study by Abbott and Faisal [67] presents an ultra-low-cost binocular three-dimensional gaze tracking system, which the authors plan to use to control wheelchair navigation or end point control of robotic arms.

#### Tongue interfaces

Tongue movement has been interfaced using electrical switches [144], Hall-effect sensors [145], pressure sensors [146], and by measuring changes in the inductance of an air-cored induction coil, by moving a ferromagnetic material attached to the tongue into the core of the coil [75, 77, 147].

**Figure 8:**
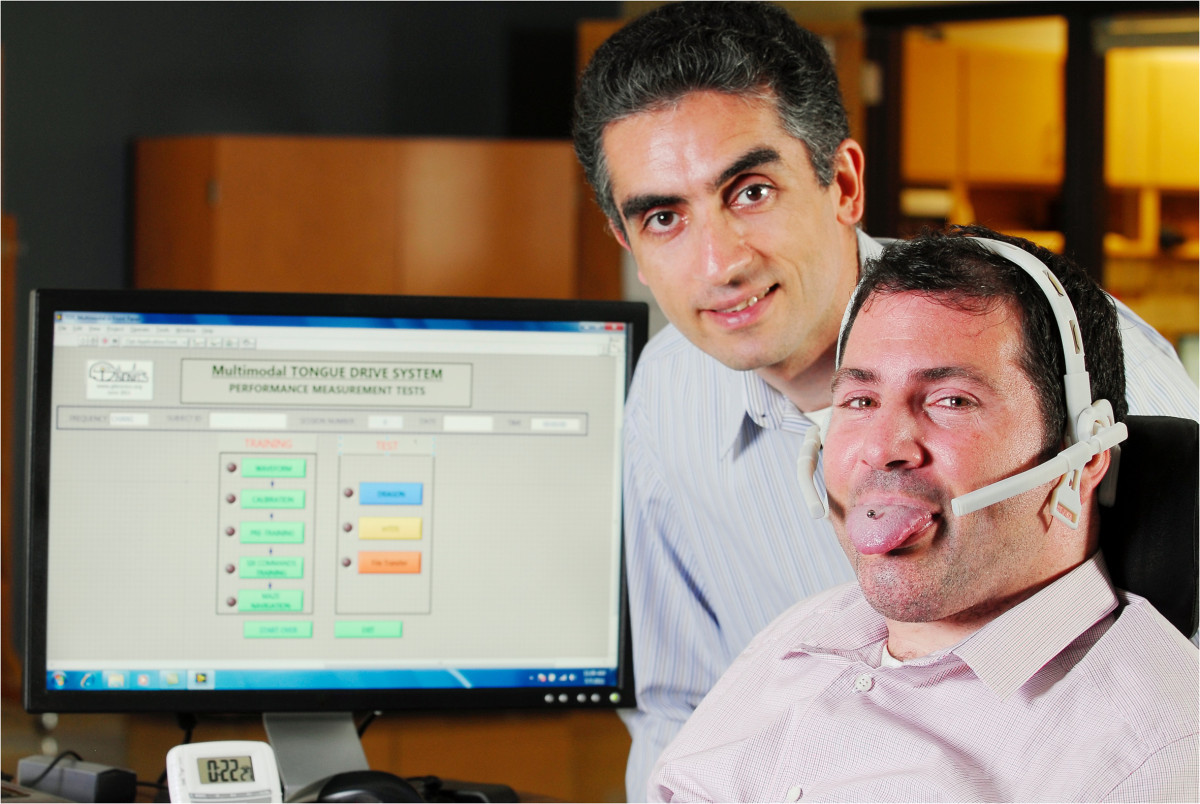
**Tongue-movement-based interfaces.** The tongue-movement-based interface developed by [76]. This system has two inductive coils mounted at the lateral parts of the mouth and a ferromagnetic material attached at the tip of the tongue. The users generate control commands by moving the tongue to one of the user-defined locations. Tongue movement interfaces take advantage of the fact that highly paralyzed patients generally have tongue control and they can move it very rapidly and accurately within the oral space. Additional file 6 shows a SCI patient performing a driving task with a wheelchair that is controlled with this tongue-movement-based interface. Figure courtesy of Dr. Maysam Ghovanloo.

The tongue-movement-based control interface developed by the research group of Prof. M. Ghovanloo at the GT-Bionics Lab (Georgia Institute of Technology, USA) is a wireless, unobtrusive, and wearable assistive technology that enables SCI patients to control computers and powered wheelchairs [75, 76] (see Figure 8 and Additional file 6). This system has an inductive coil mounted at the lateral part of the mouth and a ferromagnetic material attached at the tip of the tongue. The users generate control commands by moving the tongue to one of the user-defined locations, such as touching a particular tooth with the tongue’s tip. This control interface was tested in 13 high-level SCI patients during a navigation task with a powered wheelchair. The study reported that the subjects were able to perform the experimental task with 82 percent accuracy [148].

Additional file 6: **Tongue-movement-based interface.** A men with SCI performing a driving task with a wheelchair that is controlled with the tongue-movement-based interface developed by [76]. Video courtesy of Dr. Maysam Ghovanloo. (MP4 8 MB)

The tongue interface developed by Struijk et al. [77] integrated the induction coils under the palate, where 18 sensors allowed real-time proportional control of both speed and direction similar to a conventional joystick. The system’s functionality was demonstrated in a pilot experiment with one healthy subject, where a typing rate of up to 70 characters per minute was obtained with an error rate of 3 percent. Recently, two alternative sensor designs based on the previously described system have been proposed in order to reduce the size of the sensor pad and increase the comfort of the oral interface [149].

#### Head interfaces

Head movements are generally measured using accelerometers and used to control powered-wheelchairs [70–72, 150, 151]. The direction of the head inclination controls the wheelchair’s direction and the velocity of the wheelchair is proportional to the inclination angle. Artificial neural networks are usually implemented in the control interface to detect with higher accuracy the movement intention of the user [70]. However, all the research studies found were tested with healthy subjects, which does not provide reliable evidence of its actual usability in patients with severe movement impairments.

Alternative sensors include ultrasonic sensors [74] and camera-based interfaces [64, 73]. An important disadvantage of camera-based interfaces is that their functionality largely depends on the light conditions that result in the need of repetitive calibrations during the day [152].

#### Speech interfaces

In speech-based interfaces, the voice commands of the user are recorded using conventional microphones and translated into control signals through speech-recognition algorithms. Generally, speech-recognition requires training, which consists of the recording of the voice commands and their subsequent manual classification.

Fan and Li [78] developed a speech-based interface for the control of an upper-extremity prosthesis that could recognize 15 different voice commands with an accuracy of 96 percent. Speech has been also used to control powered wheelchairs [79] or external robotic arms [80]. The main drawback of speech-based interfaces is their high sensitivity to ambient noise, which compromises signal detection and classification. The recognition accuracy of the speech-based interface developed by Lv et al. [153] for the control of a robot was decreased by 30 percent when ambient noise was present.

#### Hand interfaces

Hand joysticks are generally implemented for the control of powered wheelchairs [82] and external robotic arms [83, 154]. The study by Johnson et al. [81] controlled the end-point position of a five-DOF upper-extremity orthosis with a joystick operated with the contralateral hand.

#### Other interfaces with parallel systems

Some of the control interfaces described previously in sections *Interfacing with the Plant* and *Interfacing with the Actuators* have also been used to measure signals from parallel systems. For instance, EMG signals measured from muscles of the upper-extremity have been used to control electrically powered wheelchairs [155–157]. Another example are the force-based interfaces used to control power-assisted wheelchairs [158] in which the wheelchair detects and amplifies the force applied by the user’s arm on the wheel.

### Hybrid control interfaces

Each of the methods described in this review present unique capabilities and limitations. A recent approach to take advantage of this functional heterogeneity is the development of *hybrid* control interfaces, which fuse data from several sensors. The concept behind hybrid control interfaces is to exploit the advantages and diminish the limitations of each individual system by combining them. Hybrid approaches can improve accuracy, reliability, and robustness compared to individual use of control interfaces [159]. Moreover, hybrid control interfaces have the potential advantage of adapting better to the needs and capabilities of the user since they can rely on several information sources. For instance, if the user gets fatigued and EMG signals present poor quality, the hybrid system could adapt and give priority to other information sources. EEG-EMG-based control interfaces are an example of a hybrid system that has been extensively used to operate active prostheses, orthoses, and robotic wheelchairs [159]. In active lower-extremity prostheses, hybrid interfaces combine EMG and mechanical inputs to identify the phase of the gait cycle and actions like sitting or standing [160–162]. Other examples include the combination of EMG with foot switches [163], IMUs [164] and video cameras [165].

### Evaluation of control interfaces

While there is a large variety of control interfaces, a small number of studies have focused on their formal performance evaluation and comparison [166–173]. The performance of control interfaces is rarely evaluated consistently, which prevents an objective evaluation and comparison. The information provided in most cases is insufficient to determine the advantages and limitations of a given control interface compared to the current state of the art. A significant step toward the standardization of outcome measures has been made by the American Academy of Orthotics & Prosthetics, which provides comprehensive recommendations for the evaluation of upper-limb prostheses [174].

A better understanding of the limitations and capabilities of the different control interfaces, through objective and quantitative evaluations during functional tasks, can provide relevant information for the selection of the most suited control interface for a specific application. One-dimensional screen-based position-tracking task experiments [166, 173] have been used to evaluated the performance of EMG-, force-, joystick- and wrist angle-based control interfaces in terms of tracking error, information transmission rate, human-operator bandwidth or crossover frequency, and effort. Guo et al. [167] compared SMG-, EMG-, force- and wrist angle-based interfaces during a series of screen-based discrete tracking tasks with and without a simultaneous auditory attention task. Even though these studies do not evaluate the interface performance during functional tasks, they can provide insight on their potential value as control interfaces for active movement-assistive devices. Other common evaluation methods are tests based on the Fitts’ Law paradigm in two- or three-dimensional space to compare the performance of different control strategies [168, 172, 175]. Recently, Simon et al. [176] developed the Target Achievement Control test, a real-time virtual test environment that closely resembles reality and allows the evaluation of upper-extremity movement control of multifunctional prostheses. The Target Achievement Control test is being used by several research groups to compare the performance of different control algorithms [177, 178].

### Physiological and artificial learning

A common issue in all of the interfacing modalities described in this review is the fact that the user must learn how to use the control interface. Artificial learning is an alternative approach that aims at relieving the user from the training period by rapidly identifying individualized signal patterns that are associated to a specific intention. Casadio et al. [179] developed a control interface for highly paralyzed patients optimizing a subject-specific mapping of residual voluntary movements with control inputs for assistive devices. In practice, this approach is translated into a mutual adaptation in which both user and assistive device learn from each other [90]. Several studies have shown that including artificial learning in BCIs accelerates the adaptation process [11, 180, 181]. Artificial learning/intelligence has also been used for the development of shared-controlled algorithms that aim to combine the intelligence of the human and the assistive device to reduce the concentration effort [90, 182]. With this approach, low-level functions are controlled by the assistive device, and the role of the user is reduced to only give high-level commands, supervise and fine tune the functioning of the system.

In addition to artificial learning, researchers have also been investigating the physiological learning capabilities of the human-controller when subjects are asked to interact with control interfaces that have intuitive and non-intuitive mappings between EMG signals and cursor or device movement. Radhakrishnan et al. [183] and Pistohl et al. [184] found that subjects could learn non-intuitive mappings with a final performance nearly equal to the intuitive mappings using both virtual and real prosthetic control environments. In addition, the recent study by Antuvan et al. [185] extended the aforementioned studies investigating the influence of previously learned mappings on new control tasks. They found that learning curves transferred across subsequent trials have the same mapping, independent of the tasks to be executed, which suggests that maximal performance may be achieved by learning a constant, arbitrary mapping rather than using the common approach of subject- and task-specific mappings.

### Design considerations

#### Intuitiveness

A control interface should be intuitive, enabling the user to operate their active movement-assistive device subconsciously (i.e. the way healthy people control their limbs) with a short training period and to think and do other things while using the device. Control interfaces may require proprioceptive feedback (i.e., EPP; [131]) in cases where sensory feedback has been lost (such as in amputees), to not only rely on visual feedback, which requires considerable mental effort [186].

The reviewed literature shows that most control interfaces are tested in laboratory environments in which users can concentrate on the experimental task with minimal distractions. However, “real-world” users must deal with much more complex situations, where mental effort cannot be entirely (or even primarily) dedicated to the control of the assistive device, as they have to interact with other people and the environment. Therefore, considering that many processes run simultaneously in the brain, it is plausible to conjecture that noninvasive-BCIs would require a considerable effort of concentration to generate those specific brain signals required for the control of an active movement-assistive device. On the other hand, peripheral signals such as force or EMG are more closely linked to the control of movement. Consequently, control interfaces using these signals would appear to be more natural and intuitive for the user than noninvasive-BCIs. Nevertheless, as previously presented in section *Physiological and Artificial Learning*, shared-control strategies can reduce the concentration effort of BCIs.

#### Information source for the control interface

A second consideration in the design of a control interface required to gain both user and clinical acceptance of active movement-assistive devices, is that the control interface should avoid the sacrifice of “useful” body functions from parallel systems (e.g. eye, head or tongue movement) for deriving the motion intention of the user. Nevertheless, signals from parallel systems can be used for “supplementary control,” such as tuning control settings or switching on and off control modalities. Note that supplementary control is used on an occasional basis and therefore, does not require continuous attention of the user and never implies the sacrifice of the parallel system functionality.

#### Response time

Another essential feature of the control interface that has a determinative effect on the performance of the assistive device is the response time or delay [187]. A tradeoff between speed and accuracy exists regarding the control delay. Large delays increase accuracy of the motion intention detection, but at the same time, decreases responsiveness (and therefore performance) of the active assistive device. Farrell and Weir [187] concluded that controller delays should be kept around 100 and 175 ms for proper control of myoelectric prostheses.

#### Robustness

The system should be robust to disturbances so that variability of sensor placement during donning, slow signal changes due to variation of environmental conditions (e.g. temperature, humidity, or light), user fatigue or external forces with low (e.g., gravity) or high frequencies (e.g. automobile vibrations), do not compromise its performance. Several researchers have identified signal stability and robustness as one of the most critical requirements of control interfaces [89, 110, 188, 189].

#### Coordination of the degrees of freedom

From a functional point of view, a control interface for active movement-assistive devices should be able to coordinate multiple DOF simultaneously in an effective way. The importance and feasibility of simultaneous control has been investigated using several control modalities such as EEG- [190], EMG- [23, 168] and head movement-based interfaces [172]. Apart from functional advantages, coordinated movements give a more natural appearance than if every DOF is controlled separately. Nevertheless, the user may also need to control an individual DOF when performing a precision task such as unlocking a door or clicking the buttons of a computer mouse.

#### Independence

Ideally, an active movement-assistive device should not require assistance from other people (e.g., caretakers or family) for the preparation of the equipment, such as installing the sensors in the correct location or calibrating the system. Control interfaces that use implantable sensors or sensors integrated in the artificial plant inherently offer a clear advantage in this respect. Most of the active prosthetic devices can be self-donned [104], and recent studies focus on simplifying the training of control methods such as pattern recognition by developing interfaces that guide the user during the training of the algorithm [191].

#### Customization

Control interfaces for active movement-assistive devices need to be customized to the characteristics of the pathology, the available physiological signals, morphology of the body, and the mechanical configuration of the device. In this respect, the control interface (and maybe the rest of the device) should be able to adapt to the changing needs and capabilities of the user. Note that changes can occur over the short term (during a day) as well as over a longer term (years) periods. The monitoring of specific biomechanical descriptors could give an indication of the user’s changes and adapt the assistive device to the new situation. Moreover clinicians could use this information to evaluate a disease’s progression.

## Conclusions

This paper presented a comprehensive review of the existing non-invasive control interfaces used to operate active movement-assistive devices. A novel systematic classification method is presented to categorize the inventory of existing control interfaces. This method is based on classifying the control interfaces depending on the source of the signal, that is, which subsystem of the HMCS is the signal coming from, which physiological phenomena is generating the signal and which sensors have been used to measure the signal. We found that the classification method could successfully categorize all existing control interfaces providing a comprehensive overview of the state of the art. The classification method also represents a framework in which new sensing modalities can be included. Each method was shortly described in the body of the paper following the same structure as the classification method.

Current non-invasive BCIs appear to be limited in speed, accuracy, and reliability for the control of active movement-assistive devices. However, researchers are improving their performance by implementing artificial learning algorithms, shared-control strategies and combining BCI with other sensor modalities. Myoelectric-based interfaces are nowadays the most common method for operating active prosthetic and orthotic devices. Advanced myoelectric-based techniques, such as TMR and muscle synergy-based algorithms, are overcoming important functional limitations of standard myoelectric control. Despite the fact that muscle contraction-based interfaces are not being implemented in today’s clinical practice, these interfaces are showing significant advancements and in the future may find their way to compete with the well-established myoelectric control. Although movements and forces are the natural way for the human to interact with the environment, interfaces based on these phenomena are very challenging to use in patients with severe movement and force limitations. Movement- and force-based control interfaces are more often implemented in rehabilitation robots where patients need to train to regain mobility and strength. Finally, control interfaces based on signals from parallel systems are generally a suitable solution to derive the movement intention of highly paralyzed patients. Recently, interfaces based on parallel systems have also been implemented in hybrid sensor modalities to expand the sources of information. In this respect, artificial learning is a promising strategy to make use of these extra sources of information and to give some degree of autonomy and adaptability to the artificial controller. In addition, a better understanding on how the human-controller works and learns will have a great impact on how control interfaces are designed.

Regarding the comparison and evaluation of the existing control interfaces, we have found that while many different control interfaces have been developed, their respective performance capabilities and limitations remain unclear. There is a need for a basic consensus on the evaluation methodology to be able to compare control interfaces and select the most suitable for a specific application. An important step toward the standardization of evaluation methods is specially being made in the field of upper-extremity prostheses by the American Academy of Orthotics & Prosthetics. Furthermore, it is clear that most of the studies presenting novel control interfaces lack tests outside the laboratory environment. In this context, it is important for future studies to validate new developments by conducting experiments with real end users in an everyday life situation.

All the reviewed control interfaces (in combination with the rest of the assistive system) are still far below the performance of the human movement control system, which inevitably hinders their user’s acceptance. However, the high rate at which these technologies are advancing is a clear indication that there is global interest in developing natural and intuitive control interfaces for active movement-assistive devices.

## Consent

Written informed consent was obtained from the patient for the publication of this report and any accompanying images.

## Electronic supplementary material

Additional file 3: **Joint-rotation-based interface.** An amputee controlling shoulder flexion-extension and internal-external rotations of an active upper-extremity prosthesis with a two-DOF joystick that measures residual shoulder motion. Video reused from [56]. (MP4 1 MB)

## Abbreviations

HMCS: Human movement control system
AMCS: Artificial movement control system
EPP: Extended physiological proprioception
TMR: Targeted muscle reinnervation
BCI: Brain computer interface
EMG: Electromyography
EOG: Electrooculographic
DOF: Degree of freedom
EEG: Electroencephalography
SMG: Sonomyography
ISO: International Organization for Standardization
MEG: Magnetoencephalography
fMRI: Functional magnetic resonance imaging
NIRS: Near infrared spectroscopy
PMG: Phonomyography
AMG: Acoustic myography
MMG: Mechanomyography
RMS: Root-mean-square
MK: Myokinemetric
TAP: Tendon activated pneumatic
MT: Myotonic
MKT: Myokinetic
MTC: Muscle tunnel cineplasty
IMU: Inertial measurement unit
SCI: Spinal cord injury
FSR: force-sensitive resistor.
